# Alternative Complement Pathway Is Activated and Associated with Galactose-Deficient IgA_1_ Antibody in IgA Nephropathy Patients

**DOI:** 10.3389/fimmu.2021.638309

**Published:** 2021-06-10

**Authors:** Yen-Ling Chiu, Wei-Chou Lin, Kai-Hsiang Shu, Yi-Wen Fang, Fan-Chi Chang, Yu-Hsiang Chou, Ching-Fang Wu, Wen-Chih Chiang, Shuei-Liong Lin, Yung-Ming Chen, Ming-Shiou Wu

**Affiliations:** ^1^ Graduate Program in Biomedical Informatics and Graduate Institute of Medicine, Yuan Ze University, Taoyuan, Taiwan; ^2^ Department of Medical Research, Far Eastern Memorial Hospital, New Taipei City, Taiwan; ^3^ Graduate Institute of Clinical Medicine, National Taiwan University, Taipei, Taiwan; ^4^ Department of Pathology, National Taiwan University Hospital, Taipei, Taiwan; ^5^ Department of Internal Medicine, National Taiwan University Hospital, Taipei, Taiwan; ^6^ Department of Internal Medicine, E-Da Hospital, Kaohsiung, Taiwan; ^7^ Graduate Institute of Physiology, College of Medicine, National Taiwan University, Taipei, Taiwan

**Keywords:** complement, C5a, factor B, galactose-deficient IgA_1_, IgA nephropathy

## Abstract

**Background:**

Galactose-deficient IgA_1_ (Gd-IgA_1_) and alternative complement pathway activation are considered to be involved in the pathogenesis of IgA nephropathy (IgAN). Nevertheless, the relationships between alternative pathway activation and disease activity or Gd-IgA_1_ level remains unclear.

**Methods:**

Ninety-eight biopsy-diagnosed IgAN, twenty-five primary focal segmental sclerosis (FSGS) patients and forty-two healthy individuals were recruited in this study. Among them, fifty IgAN patients received immunosuppression. Follow-up blood samples at 1 and 3~6 months after immunosuppression were collected. Plasma levels of complement C5a, factor Ba and Gd-IgA_1_ were measured and analyzed. Immunostaining for complement was performed in twenty-five IgAN and FSGS patients.

**Results:**

At baseline, IgAN patients had higher levels of plasma C5a, factor Ba and Gd-IgA_1_ than control subjects. Gd-IgA_1_ levels positively correlated with plasma C5a and factor Ba. In addition, levels of factor Ba and Gd-IgA_1_ were positively associated with proteinuria and negatively associated with renal function. Immunostaining revealed positive staining for factor Bb and C3c in glomeruli in IgAN patients, but not in FSGS patients. At baseline, patients receiving immunosuppression had more severe proteinuria and higher factor Ba. After 6 months, eGFR declined and proteinuria persisted in patients without immunosuppression. In contrast, patients who received immunosuppression exhibited decreased plasma levels of C5a, factor Ba, and Gd-IgA_1_ as early as 1 month after treatment. Proteinuria decreased and renal function also remained stable 6 months after immunosuppression.

**Conclusions:**

Our results indicate a close relationship between alternative complement pathway activation, Gd-IgA_1_ concentration and clinical severity of IgAN. Level of complement factor B may be a potential marker for disease activity and therapeutic target in IgAN patients.

## Introduction

IgA nephropathy (IgAN) is the most frequent primary glomerulonephritis worldwide (1) with highest incidence in eastern Asia (2) and is an important cause of end-stage renal disease (3). The main pathological findings are the mesangial proliferation and dominant immunoglobulin IgA deposition in glomeruli (4, 5). Many studies revealed that aberrant glycosylation of IgA_1_ (Gd-IgA_1_) is the cause of the development of IgAN. Gd-IgA_1_ forms immune complexes with other circulatory antiglycan autoantibodies. These immune complexes subsequently deposit in renal glomeruli, initiate tissue inflammation, structural destruction and ultimately renal failure (6, 7).

The formation of immune complexes could activate the complement system which is composed of classic, alternative and lectin pathways. The activation of complement system in IgAN were initially demonstrated by the presence of complement component C3, C4d, and C5-9 in glomeruli in IgAN patients (8–10). In recent years, researchers started to explore which complement pathway mediates the Gd-IgA_1_/anti-glycan immune complex induced glomerular damage in IgAN patients. Classical pathway is not considered to participate in this process, as C1q, a key indicator of classical pathway activation, was not usually found in glomerular specimens in IgAN patients (11). Several studies revealed the involvement of lectin pathway in IgAN, such as the deposition of manos binding lectin (MBL) (12, 13), L-ficolin, MBL-associated protease 1~3, (MASP-1~3) (13). The deposition of C3 is not adequate to indicate alternative pathway involvement because C3 is also involved in the activation of classical pathway. There is only one study demonstrating properdin glomerular deposition in 30~90% IgAN patients which could specifically indicate the activation of alternative pathway (14). Other indirect evidences include the association of complement factor H-related protein or complement factor H gene with IgAN incidence (15–17). More evidences are necessary to clarify the role of alternative pathway in IgAN.

In this study, we investigated the blood levels of alternative/terminal complement factor B/C5a and tissue levels of factor Bb in IgAN patients. Their levels were correlated with clinical severity and the concentration of Gd-IgA_1_. The changes of these molecules after immunosuppression were also evaluated. The results demonstrated the role of the alternative complement pathway in IgAN.

## Materials and Methods

### Patients and Blood Sample Collection

Patients aged between 20~80 years old who received renal biopsy during January 2015 to December 2019 in National Taiwan University Hospital and diagnosed as IgAN were recruited into this study. Besides IgAN patients, 42 age-matched healthy subjects and 25 primary focal segmental sclerosis patients were also included as control subjects. Patients with inadequate laboratory data records and diagnosed as secondary IgAN were excluded. All biopsies were performed before immunosuppression and blood samples were collected during the admission for renal biopsy before the initiation of immunosuppression. All blood samples were collected in test tubes containing EDTA, processed immediately and stored at -80°C before the measurement of antibody titer and complement concentration. Among the 98 included IgAN patients, 50 patients received immunosuppression and follow-up blood samples were obtained in 17 and 27 of these patients 1 and 3~6 months after the initiation of immunosuppressive treatments, respectively. The indication of immunosuppression was based on the judgement of primary care nephrologists which included nephrotic range proteinuria, significant deterioration of renal function or prominent increase of proteinuria during a short follow-up period. The immunosuppressive treatments were systemic oral corticosteroid alone or in combination with alkylating agents (N=5), cyclosporine (N=5) while four patients also received intravenous pulse steroid. Informed consents were obtained from all participants or their legal representatives. This study was approved by the Institutional Review Board of National Taiwan University Hospital and was performed in compliance with the Declaration of Helsinki.

### Clinical Parameters

Demographic characteristics, including age, sex, hepatitis, malignancy, diabetes, hypertension and use of angiotensin converting enzyme inhibitor/angiotensin II receptor blocker (ACEi/ARB) were recoded. Levels of serum creatinine, serum albumin, total cholesterol, estimated glomerular filtration rate (eGFR, calculated using the Chronic Kidney Disease Epidemiology Collaboration equations formula for Taiwanese adults) (18) and the magnitude of urine protein loss (urine protein to creatinine ratio, UPCR) before biopsy were recorded. Among 50 patients received immunosuppression, follow-up serum creatinine and UPCR along with measurement of follow-up Gd-IgA_1_, C5a and factor Ba levels were obtained 1 and 3~6 months after immunosuppression was initiated.

### Measurement of Plasma Gd-IgA_1_, C5a, and Factor Ba

Plasma Gd-IgA_1_ was measured with enzyme linked immunosorbent assay (ELISA) (Immuno-Biological Laboratories, Minneapolis, MN, USA). Plasma levels of C5a and factor Ba were measured with C5a ELISA kit (Aviva Systems Biology, San Diego, CA, USA) and the MicroVue Ba fragment EIA kit (Quidel Corporation, San Diego, CA, USA). All assays were performed using the protocols provided by the manufacturers.

### Immunofluorescence Staining in Kidney

Frozen specimens of kidney biopsy were obtained from 25 IgAN and 25 FSGS patients and were sectioned into 4-μm thick slices. For factor Bb staining, tissue sections were incubated with mouse anti-human complement factor Bb antibody (Bio-Rad, UK). After washing, tissue sections were incubated with the secondary fluorophore-conjugated goat anti-mouse secondary antibody (Abcam, Cambridge, UK). Tissues were then mounted and subjected to fluorescence microscopy. For C3c staining, tissue sections were incubated with FITC conjugated polyclonal rabbit anti human C3c complement antibody (Dako, Denmark). The intensity was graded into 1+ ~ 4+ according to the fluorescence intensity.

### Statistical Analysis

For baseline characteristics, continuous variables with normal distribution were expressed as mean ± standard deviation and those that were not normally-distributed were expressed as median (interquartile range). Differences among groups were compared using Kruskal-Wallis multiple comparison test because more than three groups were compared and data were not assumed to be Gaussian’s distribution. Paired data was compared using the Wilcoxon matched-pairs signed rank test. Categorical variables were expressed as frequency and percentages and were compared using Fisher exact test. Relationships between two continuous variables were analyzed using Pearson correlation. *P* value less than 0.05 was considered as statistically significant. Statistical analyses were performed with Prism software, version 8 (GraphPad Software, San Diego, CA, USA).

## Results

In total, we included 98 primary IgAN patients in this study. The average serum creatinine level was 1.68 ± 1.29 mg/dL with an average eGFR of 55.22 ± 34.13 mL/min. Most patients had significant proteinuria with an average UPCR of 2.78 ± 3.14 g/g. (**Table 1**) These results indicated that our patients in this study already had significant renal damage when inclusion.

**Table 1:** Baseline characteristics of all IgAN patients.

	Total (N = 98)
Age (years)	45.11 ± 13.60
Male (%)	46 (46.9%)
Diabetes (%)	7 (7.1%)
Hypertension (%)	43 (43.8)
Use of ACEI/ARB (%)	63 (63.3%)
UPCR (g/g)	2.78 ± 3.14
Creatinine (mg/dL)	1.68 ± 1.29
eGFR (ml/min/1.73m^2^)	55.22 ± 34.13
Gd-IgA_1_ Ab titer (ng/mL)	7920 (5129-11708)
C5a (ng/mL)	3476 (1095-4913)
Factor Ba (ng/mL)	2865 (1425-4379)

Normally-distributed continuous variables including age, UPCR, creatine, eGFR are expressed as mean ± standard deviation. Non-normally-distributed continuous variables such as Gd-IgA1 Ab titer, C5a and Factor Ba level are expressed as median (interquartile range).

### Alternative Complement Pathway Is Activated in IgAN Patients and Positively Correlated With the Disease Activity

**Figure 1:**
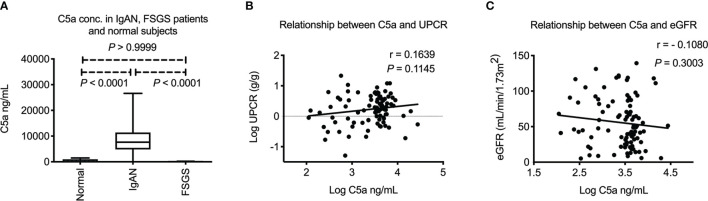
Plasma C5a in IgA nephropathy patients. **(A)** Plasma C5a concentration was significantly higher in IgAN patients than FSGS patients or healthy subjects (both *P* < 0.0001). There was no difference of plasma C5a between FSGS patients and normal subjects. (*P* > 0.9999) **(B)** Plasma C5a level did not correlate with level of proteinuria in IgAN. **(C)** Plasma C5a level did not correlate with renal function. UPCR, urinary protein to creatinine ratio. eGFR, estimated glomerular filtration rate. FSGS, focal segmental glomerulosclerosis.

As mentioned above, formation of immune complex is known to activate the complement pathway. C5 is the key mediator that regulates the final common complement pathway, generating C5a, the end product of complement activation that can be detected in circulation. We examined the plasma C5a levels among all study participants. As shown in **Figure 1A**, plasma C5a was increased in IgAN patients when compared to FSGS patients or normal subjects (both *P* < 0.0001). There was no difference in levels of plasma C5a between FSGS patients and normal subjects (*P* > 0.9999). These results indicated that the increase of plasma C5a was specific to IgAN patients but not FSGS patients or normal subjects. The level of plasma C5a tended to be positively correlated with proteinuria, but there was no statistical significance (**Figure 1B**). In addition, there was no correlation between plasma C5a and eGFR (**Figure 1C**).

**Figure 2:**
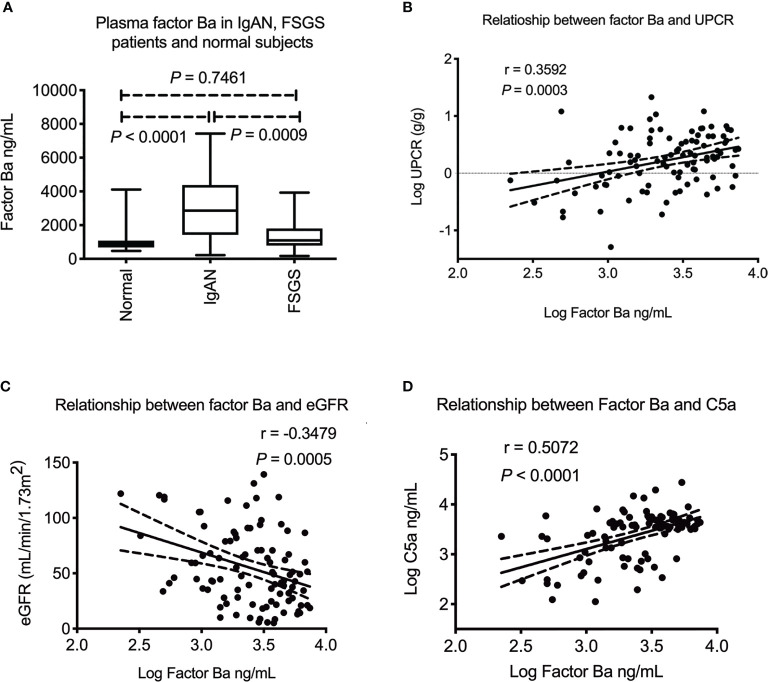
Plasma factor Ba in IgA nephropathy patients. **(A)** Plasma factor Ba concentration was significantly higher in IgAN patients than FSGS patients or healthy subjects (*P* = 0.0009 and *P* < 0.0001) There was no difference of plasma factor Ba between FSGS patients and normal subjects. (*P* = 0.7461). **(B)** Plasma factor Ba level significantly correlated with level of proteinuria in IgAN patients (*P* = 0.0003, r = 0.36). **(C)** Plasma factor Ba level negatively correlated with renal function (*P* = 0.0005, r = -0.35). **(D)** There was positive association between level of plasma factor Ba and C5a (*P* < 0.0001, r = 0.51). UPCR, urinary protein to creatinine ratio; eGFR, estimated glomerular filtration rate; FSGS, focal segmental glomerulosclerosis.

Upstream of C5a, factor B is the key mediator that activates alternative pathway and factor Ba is the product of alternative complement pathway activation which can be detected in circulation. We next examined the plasma level of factor Ba. As shown in **Figure 2A**, plasma factor Ba was increased in IgAN patients when compared to FSGS patients or normal subjects (*P* = 0.0009 and *P* < 0.0001, respectively). There was no difference in levels of factor Ba between FSGS patients and normal subjects. (*P* = 0.7461) These results indicated that the increased level of plasma factor Ba was specific to IgAN patients but not FSGS patients or normal subjects. Furthermore, the level of plasma factor Ba was positively correlated with the degree of proteinuria (*P* = 0.0003, r = 0.36, **Figure 2B**) and negatively correlated with eGFR (*P* = 0.0005, r = -0.35, **Figure 2C**). Factor Ba level also positively correlated with the level of C5a concentration (*P* < 0.0001, r = 0.51, **Figure 2D**). These results may indicate that, in contrast to the terminal pathway, activation of alternative pathway as measured by level of Ba, positively correlated with clinical severity and the subsequent activation of terminal complement pathway in IgAN patients.

**Figure 3:**
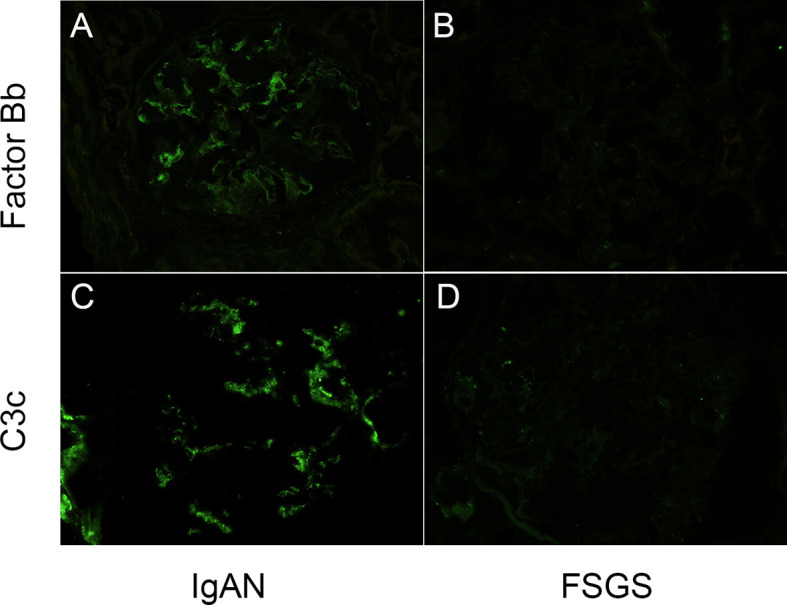
Glomerular complement factor Bb and C3c deposits were detected in IgAN patients. **(A, B)** Mesangial positivity can be seen in the complement factor Bb and C3c stains on the glomerulus of a patients with IgA nephropathy. **(C, D)** No intensity can be detected in the complement factor Bb and C3c stains on the glomerulus of a patient with focal segmental sclerosis. (Magnification: 400X).

To demonstrate the local activation of alternative complement pathway in IgAN patients, renal biopsy specimens of twenty-five IgAN patients and twenty-five FSGS patients were obtained for factor Bb and C3c immunofluorescence staining. The staining of factor Bb in glomeruli was positive in IgAN patients (**Figure 3A**); 1+~2+ in thirteen (52%) and trace to 1+ staining in seven patients (28%). In contrast, the staining of factor Bb was negative in all FSGS patients (**Figure 3B**). To evaluate the activation of the subsequent complement pathway, C3c was also examined in biopsy specimens. Grade 3~4+ staining was seen in all IgAN patients but was negative for all FSGS patients (**Figures 3C, D**). The result indicated alternative and subsequent complement pathways were activated in most, if not all IgAN patients.

### Gd-IgA_1_ Is Increased in IgAN Patients and Correlated With Disease Activity and Complement Activation

**Figure 4:**
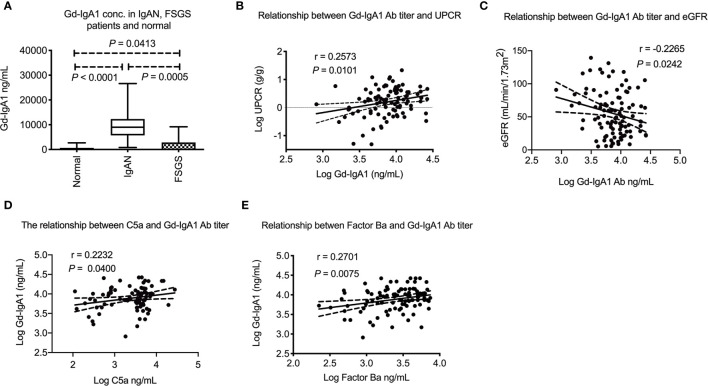
Plasma Gd-IgA_1_ level in IgA nephropathy patients and their relationship with plasma C5a and factor Ba. **(A)** Plasma Gd-IgA_1_ concentration was significantly higher in IgAN patients than FSGS patients or healthy subjects (*P* = 0.0005 and *P* < 0.0001, respectively). The Gd-IgA_1_ level was only a litter higher in FSGS patients than normal subject (*P* = 0.0413). **(B)** Plasma Gd-IgA_1_ level was positively correlated with level of proteinuria (*P* = 0.0101, r = 0.26). **(C)** Plasma Gd-IgA_1_ level was negatively correlated with renal function (*P* = 0.0242, r = -0.23). **(D)** Positive association between level of plasma C5a and Gd-IgA_1_ (*P* = 0.0400, r = 0.22). **(E)** Positive association between level of plasma factor Ba and Gd-IgA_1_ (*P* = 0.0075, r = 0.27). UPCR, urinary protein to creatinine ratio; eGFR, estimated glomerular filtration rate.

The median plasma Gd-IgA_1_ concentration among IgAN patients was 7920 (5129–11708) ng/mL, which was much higher than FSGS patients and normal subjects. (P = 0.0005 and *P* < 0.0001, respectively, **Figure 4A**). The Gd-IgA_1_ concentration was a litter higher in FSGS patients than normal subjects which might be due to chronic kidney disease. (P = 0.0413) Plasma Gd-IgA_1_ concentration was also positively correlated with the degree of proteinuria (*P* = 0.0101, r = 0.26, **Figure 4B**) and negatively correlated with eGFR (*P* = 0.0242, r = -0.23, **Figure 4C**). Interestingly, plasma Gd-IgA_1_ concentration positively correlated with plasma C5a level (*P* = 0.0400, r = 0.22, **Figure 4D**) and factor Ba (*P* = 0.0075, r = 0.27, **Figure 4E**). These phenomena indicate that plasma Gd-IgA_1_ has a significant correlation with the activation of both alternative and terminal complement pathways.

### Decrease of Gd-IgA_1_ Titer and Decreased Activation of Alternative Complement Pathway Were Observed After Immunosuppression

**Figure 5:**
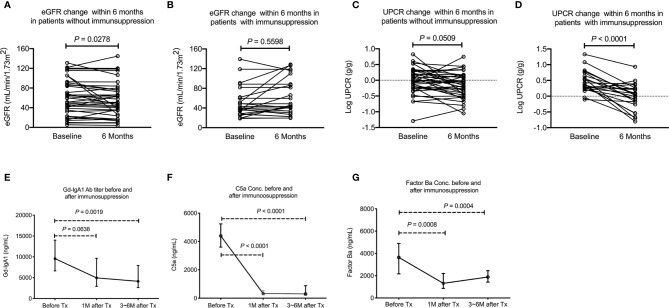
Decreased activation of alternative and terminal complement pathway and stabilized renal function after immunosuppression. **(A)** eGFR decreased slightly among patients who did not receive immunosuppression (*P* = 0.028). **(B)** No change in eGFR among patients received immunosuppression. **(C)** UPCR tended to decrease slightly among patients who did not receive immunosuppression (*P* = 0.051). **(D)** UPCR decreased significantly in patients who received immunosuppression (*P* < 0.0001). **(E)** Plasma Gd-IgA_1_ level tended to decrease 1 months after immunosuppression (N = 17, *P* = 0.0638) and 3~6 months after immunosuppression (N = 20, *P* = 0.0019). **(F)** Plasma C5a significantly decreased 1 (N = 17, *P* < 0.0001) and 3~6 months after immunosuppression (N = 20, *P* < 0.0001). **(G)** Plasma factor Ba level significantly decreased 1 (N = 17, *P* = 0.0008) and 3~6 months after immunosuppression (N = 20, *P* = 0.0004). UPCR, urinary protein to creatinine ratio; eGFR, estimated glomerular filtration rate.

Among the 98 IgAN patients, fifty patients received immunosuppression based on the clinical judgement made by the primary care nephrologists after renal biopsy. All 50 patients received oral systemic steroid therapy in whom 5 patients received a combination of steroid with cyclosporine and another 5 patients received steroid with cyclophosphamide. To explore the change of plasma C5a, factor Ba and Gd-IgA_1_ concentration after immunosuppression, blood samples were collected in 17 and 27 patients 1 and 3~6 months after the initiation of immunosuppression after renal biopsy, respectively. No follow up data was available in patients without immunosuppression because blood samples were not collected. Baseline clinical data, Gd-IgA_1_, C5a and factor Ba concentration were compared between patients without immunosuppression (N = 48) and with immunosuppression with follow up blood sample (N = 27). Patients who received immunosuppression and having follow up blood samples had more severe proteinuria and tended to have worse renal function before renal biopsy than the patients who didn’t receive immunosuppression. The initial Gd-IgA_1_ concentration was not different between patients who received or didn’t receive immunosuppression. However, the initial plasma factor Ba was higher (*P* = 0.003) and C5a (*P* = 0.068) also exhibited a trend to be higher in patients receiving immunosuppression. (**Table 2**) Importantly, renal function (eGFR) decreased slightly 6 months after renal biopsy in patients who did not receive immunosuppression (**Figure 5A**, eGFR 59.9 decreased to 54.9, *P* = 0.028) while renal function remained stable in patients who received immunosuppression (eGFR 50.9 increased to 60.7, *P* = 0.560, **Figure 5B**). Proteinuria tended to decrease slightly 6 months after renal biopsy in patients who didn’t receive immunosuppression (*P* = 0.051, **Figure 5C**) while it was significantly decreased 6 months after immunosuppression in patients who received immunosuppression (*P* < 0.0001, **Figure 5D**). One month after immunosuppression, plasma Gd-IgA_1_ concentration tended to decrease in patients receiving immunosuppression. (*P* = 0.0638, **Figure 5E**) Concentrations of plasma C5a and factor Ba both decreased quickly 1 month after immunosuppression (*P* < 0.0001 and *P* = 0.0008 respectively, **Figures 5F, G**). At 3~6 months after immunosuppression, Gd-IgA_1_ was significantly lower than the level before immunosuppression (*P* = 0.0019, **Figure 5E**) along with levels of factor C5a and factor Ba. (*P* < 0.0001 and *P* = 0.0004 respectively, **Figures 5F, G**).

**Table 2:** Comparisons of baseline characteristics among IgAN patients with and without immunosuppression.

	without immunosuppression (N = 48)	with immunosuppression (N = 27)	
Age (years)	41.82 ± 12.40	45.75 ± 14.98	*P* = 0.3600
Male (%)	26 (54.17%)	10 (37.04%)	*P* = 0.6381
Diabetes (%)	1 (2.08%)	2 (7.41%)	*P* = 0.2462
Hypertension (%)	24 (50.00%)	11 (40.74%)	*P* = 0.4783
Use of ACEi/ARB (%)	32 (66.67%)	19 (70.37%)	*P* = 0.2637
UPCR (g/g)	1.39 ± 1.30	3.78 ± 4.21	*P* < 0.0001
Creatinine (mg/dL)	1.65 ± 1.50	1.60 ± 0.98	*P* = 0.1190
eGFR (ml/min/1.73m^2^)	64.49 ± 37.37	48.87 ± 31.70	*P* = 0.0609
Gd-IgA_1_ Ab titer (ng/mL)	7120 (3847-12163)	8512 (5228-13802)	*P* = 0.2971
C5a (ng/mL)	2348 (567-4904)	4350 (2254-5222)	*P* = 0.0683
Factor Ba (ng/mL)	2222 (990-3622)	3600 (1968-4960)	*P* = 0.0032

Normally-distributed continuous variables including age, UPCR, creatine, eGFR were expressed as mean ± standard deviation. Non-normally-distributed continuous variables including Gd-IgA1 Ab titer, C5a and Factor Ba level were expressed as median (interquartile range). UPCR, urinary protein to creatinine ratio; eGFR, estimated glomerular filtration rate; ACEi/ARB, angiotensin converting enzyme inhibitor/angiotensin II receptor blocker.

## Discussion

The formation of immune complexes induces the activation of complement pathways. In previous studies, a SNP rs6677604 representing deletion of complement factor H-related gene 1 and 3 was confirmed to have a log-additive protective effect in IgAN (16). The level of circulating factor H-related protein 1 (FHR-1), a negative regulator of the alternative pathway, negatively correlates with eGFR (19). These studies may indicate the loss of inhibition of the alternative pathway is detrimental in IgAN patients. *In vitro*, polymeric human IgA also directly activates the alternative pathway (20). To our knowledge, this is the first study demonstrating the terminal and alternative complement activation products, C5a and factor Ba, were increased in IgAN patients. Level of factor Ba also correlated with clinical disease activity, including UPCR and renal function. In contrast to patients who did not receive immunosuppression, preserved renal function and decrease of proteinuria were seen in patients receiving immunosuppression. In parallel, the clinical benefit was also associated with the decrease of complement activation product C5a and factor Ba. In addition, factor B was also detected in renal tissue from IgAN patients but not FSGS patients. All these findings indicate that the alternative complement pathway plays a crucial role in the progression of IgAN and is a valuable treatment target in IgAN patients. A clinical trial (https://clinicaltrials.gov/ct2/show/NCT03373461) using orally-administered small molecule inhibitor LPN023 targeting factor B in IgAN patients is ongoing (21).

We also found that the titer of Gd-IgA_1_ has positive correlation with UPCR and negative correlation with eGFR in IgAN patients. Suzuki et al. also reported that the disease activity of IgAN assessed by hematuria and proteinuria correlated with serum levels and changes of Gd-IgA_1_ and IgA/IgG immune complexes (22). In contrast, Zhang et al. did not find that there was any correlation between Gd-IgA_1_ antibody and eGFR or proteinuria (23). When compared to Zhang’s study, our patients had more severe proteinuria (daily urine protein loss: 2.78 ± 3.14 *vs.* 1.80 ± 1.71) and less renal reserve (eGFR: 55.22 ± 34.13 *vs.* 92.84 ± 33.57). This phenomenon may indicate that the Gd-IgA_1_ antibody titer has more clinical influence in moderate to severe IgAN patients than patients with mild disease activity. Our findings are compatible with previous studies that the level of circulatory Gd-IgA_1_ positively correlates with advanced pathological findings and future renal function decline (24, 25). It is interesting that our results revealed that the levels of alternative complement pathway activation product, factor Ba and C5a, were positively correlated with the concentration of Gd-IgA_1_. Higher Gd-IgA_1_ may lead to more anti-Gd-IgA_1_ antibody/Gd-IgA_1_ immune complex formation, then increased alternative complement pathway activation leading to the accumulation of increased activation products, factor Ba and C5a, as seen in our patients.

In this study, Gd-IgA_1_ decreased after immunosuppression. Although the underlying mechanism of generation and regulation of Gd-IgA_1_ in IgAN patients is unclear, it is believed that inflammatory mediators play an important role. Suzuki et al. found IL-6 and leukemia inhibitory factors increased the production of Gd-IgA_1_ in B cells from IgAN patients but not controls (26). In IgA_1_-producing cells from patients with IgAN *vs.* healthy controls, IL-6 showed increased and prolonged activation of STAT3. Inhibition of STAT3 signaling reduces IgA_1_ autoantigen production in IgAN patients (27). It is, therefore, reasonable to speculate that the immunosuppression may decrease the generation of Gd-IgA_1_ in IgAN patients. In a study involving fourteen IgAN patients, significantly reduction of proteinuria and the levels of serum IgA, Gd-IgA_1_ determined by anti-human IgA(ab’)_2_ then biotinylated *Sambucus nigra* lectin, and IgA-IgG immune complexes was seen after prednisolone therapy (28). The decrease of Gd-IgA_1_, therefore anti-Gd-IgA_1_ Antibody/Gd-IgA_1_ immune complex, was associated with the decrease of complement activation product indicating it is probable that decreasing Gd-IgA_1_ by immunosuppression could attenuate the activation of alternative complement pathway in IgAN patients.

As shown in our study, the product of activation of alternative complement, such as factor Ba, correlates with disease activity and is decreased after immunosuppression. Whether these markers help to decide the use of immunosuppression is an important issue. Further studies to analyze whether these markers are good predictors of immunosuppressive response is necessary. Similar issue would be whether IgAN patients with higher alternative complement activation end products, such as factor Ba, levels are the preferred candidates for alternative complement pathway inhibition. Besides, it may be useful to determine if levels of factor Ba can be used for the monitoring of the effect of immunosuppression. Our results revealed that the decrease of these complement activation products happened as early as one month after immunosuppression. To analyze the use of these markers in predicting renal outcome, larger and longer clinical studies are necessary.

There were several limitations in this study. First, whether KM55 antibody is specific to Gd-IgA_1_ may require additional research to confirm. Yamasaki et al. showed that the epitope of this antibody was the PST(Gal-NAc)PP motif (29). The galactose-deficient form of O-glycan at Thr233 is more frequently observed than that at Thr225 in serum IgA_1_ isolated from healthy control and IgA_1_ myeloma protein (30). This indicated that serum Gd-IgA_1_ detected by KM55 antibody predominantly contains P231ST(Gal-NAc)PP235 sequence. In this study, the close relationship between clinical severity and titer of Gd-IgA_1_ also implicates that there is a significant specificity against Gd-IgA_1_ by this antibody. Secondly, anti-Gd-IgA_1_ antibody also plays an important pathogenic role in IgAN, because anti-Gd-IgA_1_ antibody complexes with Gd-IgA_1_ forming immune complexes which activate complement pathways. However, we did not measure the level of anti-Gd-IgA_1_ antibody because there was yet no commercial kit available. Thirdly, our immunosuppressant treatment was not randomized and thus the observed beneficial effects of treatment might be subjective to selection bias. Finally, we did not investigate the level of Gd-IgA_1_ and complement proteins with longer follow-up duration, and whether complement protein levels increase after stopping treatment requires further studies. The last limitation is that we can’t conclude the decrease of Gd-IgA_1_, C5a and factor Ba after immunosuppression is due the immunosuppressive effect, since we don’t have these data in patients without immunosuppression. However, we can expect there will be no prominent change of the de-glycosylated antibody or complement activation product during short time period (maybe within half a year) since no treatment change was conducted in patients without immunosuppression.

In conclusion, our results revealed the close positive relationship between alternative complement activation product, factor Ba and C5a, and the disease activity and concentration of Gd-IgA_1_. Our results indicate the probability of the mechanistic link of alternative pathway complement in disease pathogenesis and suggest the future research direction of targeting factor B in the treatment and monitor of IgAN

## Data Availability Statement

The original contributions presented in the study are included in the article/supplementary material. Further inquiries can be directed to the corresponding author.

## Ethics Statement

The studies involving human participants were reviewed and approved by the Institutional Review Board of National Taiwan University Hospital. The patients/participants provided their written informed consent to participate in this study

## Author Contributions

C-YL acquired data, performed data analysis and manuscript preparation. L-WC performed immunostaining, data analysis and manuscript preparation. S-KH contributed to data acquiring. F-YW performed ELISA analysis. C-FC, C-YH, and W-CF helped patients recruiting. C-WC designed the study, analyzed data, patients recruiting, raised fund and prepared manuscript. L-SL, C-YM, and W-MS edited and corrected the manuscript. All authors contributed to the article and approved the submitted version.

## Funding

This study was supported by research grants from Mrs. Hsiu- Chin Lee Kidney Research Foundation, National Taiwan University Hospital (107-S3759 and 109-S4556), Far Eastern Memorial Hospital (FEMH-2019-C-023), and Ministry of Science and Technology, Taiwan (MOST-108-2314-B-002-053).

## Conflict of Interest

The authors declare that the research was conducted in the absence of any commercial or financial relationships that could be construed as a potential conflict of interest.
